# The Dose*From the *American Homæopathic Review*, December, 1864. Republished by request.

**Published:** 1888-12

**Authors:** P. P. Wells

**Affiliations:** Brooklyn, N. Y.


### THE DOSE.*

P. P. Wells, M. D., Brooklyn, N. Y.

Correspondence.—"*Dear Sir:* Your articles in some back volumes of the *Review*, upon diarrhœa, dysentery, rheumatism, pneumonia, and typhoid fever have claimed my careful and repeated study, and have given me a *great deal* of satisfaction. Will you do me the great favor of supplying what seems to me to be the only omission by informing me what potencies you have fixed upon as the best in each of these diseases? Every man must indeed decide this question for himself, but it is a graver matter than any merely scientific question, for it has to do with human life, and what others have thought and done is a great element in the decision. Every physician should settle the matter as soon as possible for himself, that he may feel that he is doing the best that can be done for his patient, particularly in those diseases, as typhoid fever, diphtheria, etc., where death must be met and conquered. By the way, will you not do the profession the favor of adding to those articles soon, one upon diphtheria?

"Respectfully yours,

"__________."

---

* From the *American Homœopathic Review*, December, 1864. Republished by request.

The "omission" was not an accident. In writing practically on the diseases named above, the object was to deal with general principles and their practical application to the treatment of the sick, rather than to exhibit the writer's method for imitation. The dose was left in silence, not because unimportant, nor from a want of opinion on the part of the writer as to the principles involved. One reason for this omission was his conviction of the little value which can attach to the example of any man in this matter of the dose, both as to quantity (or potence) and repetition, aside from the principles which underlie and dominate the whole subject. Aside from these, it was of little consequence what he had done. He did not forget that in all the public discussions of this subject which he had witnessed, the burden of them had been, that the speaker had in given circumstances done so or so, and that such had been the result, and that this had been repeated by the different speakers to the end, and nothing was nearer settled, as to principles, when they were through than when they began. The interest of the whole from the beginning to the end was in the knowledge that these gentlemen had done what they said they had, and for the doing of which they had given no reason founded on any principle in nature, and that the doing was followed by the declared result. It is possible that some master-mind might from a sufficient number of such loosely observed and related facts, deduce some general principles by and by. It is certain they have very little practical value except as materials for such a generalization. The controversial writings on the subject have not a much better, or a very different, result to show. It can hardly be said of them that they have settled any one principle which all are ready to receive, and from which all may the better advance to the discovery and establishment of others, till the vexed question shall be decided. The statement of the practice and experience of the writer of these papers, as to the dose, would only add another example of this almost worthless testimony.

Another reason for the omission was the known fact of the wide difference of opinion, on this matter of the dose, entertained by members of the profession, and each claiming to be earnest and honest in his own, each relying on his own experience to sustain the preference which he declares. And, further, that this difference is not unfrequently accompanied by so much prejudice in favor of such opinion, that other opinions can hardly be fairly examined or judged. That there is a peculiar sensitiveness with many on this matter of the dose, which will hardly tolerate good-naturedly even the mention of opinions, or listen complacently to the statements of experience which differs from their own. There is certainly no reason why this should be the case, but so it is. The explanation of the singular fact is not difficult. It is sufficient to say, that a similar irritability is not found in relation to different opinions and experiences on other subjects, when these are founded on known facts and principles. In view of this state of things, it was thought as well to leave the dose to a future occasion, as to state facts to which prejudice would not listen, or to add to the sum of individual experiences, which have hitherto settled so little.

Another reason for this omission was the desire of the writer to gain, as far as possible, the attention of the profession to the principles he advanced, and to the practical application of them he recommended. He was unwilling to risk aught of success in this by mixing with them, in any degree, a matter on which he knew there existed far more of prejudice than knowledge, and on which he oftener met earnest effort to sustain or defend prejudice than to add to knowledge. This he felt to be, to too great an extent, true of all parties. For however much good men and true may regret the fact, it is true that parties do exist on this subject, and that there is met in its discussions, quite too often, more of the spirit of party than of true philosophy. Is it not a little strange that this should be so? For what has party to do with a matter like this, which, if it have any foundation whatever, it must be of ascertained truth? This truth must exist, if at all, in the very nature of things, and not in the mere opinions or prejudices of any individual or party. And, we may add, if we are in any degree desirous of its discovery, and willing to engage in honest efforts to this end, we shall have laid aside one of the greatest impediments to success when we have wholly discarded the spirit and feeling of party. It can never be a help, but only a hindrance, in the pursuit of any truth, and that which will ultimately be found to decide this whole matter of the dose is no exception. The reason of this is obvious. The very centre and soul of party spirit is prejudice, and the first effort of prejudice, in the investigation of any party question, is to extinguish both light and eyes, and failing in this, to admit light only through its own spectacles.

Another reason for the omission was a purpose of the writer to discuss this subject of the dose in a paper devoted exclusively to its consideration. The request contained in the communication at the head of this paper has decided him to enter on that duty now. And the first remark he has to make is, that the whole matter *must* be one of *law* and not at all of mere *opinion*—of law constituted of definite principles, fixed in their character, and in no way subject to change—that they may meet the varying intelligence, opinions, or prejudices of men. Like other laws of divine enactment, they will stand a fixed truth, whether it be brought to light and made an instrument of practical good, or left in darkness with the unknown; whether it be received or rejected. In this, as with other divine laws, rejection is no repeal. As with other divine laws, rejection may be followed by consequences the responsibility of which we may well dread. If this be true, then the whole duty of practical men is to ascertain the nature of this law and comply with its requirements. Criticism of the law of God in the place of obedience is no more becoming in the material than in the moral world.

We have said this matter of the dose, in the treatment of the sick, is one of law. It can hardly require argument to prove this. It follows almost of necessity from that law of cure which we all recognize. It can hardly be otherwise than plain that that power which established the curative relationship between drug agencies and diseases, and regulated this by law, should at the same time and in the same manner determine the quantities and methods of their administration. If this be so, then the idea of our correspondent, that this matter of the dose is one which "every man must decide for himself," is strictly negatived, if by this is meant more than that each must be his own interpretor of the law, which is certainly true. But before he can interpret the law he must acquaint himself with its principles and relations. To endeavor to elucidate some of these will be the object of the remainder of this paper.

In attempting a discovery of the principles which constitute the law under consideration, we are first to get a clear view of the elements of the problem. These are of two classes—those which belong to the sick man, and those of the drug. In considering the first of these, the first important fact, which we meet at the threshold of inquiry, is that we are to deal with a state of things wholly, or in part, preternatural. The natural relationship between the organs of the body and their accustomed and appropriate stimuli is perverted. The susceptibility of these organs to impressions from these stimuli is exalted, depressed, or extinguished. It may be exalted in relation to some, even to the extent of intolerance, while depression as to others permits only the feeblest response to their impressions, others seem incapable of exciting any living perception of their presence. In addition to these there are new susceptibilities to impressions from other external agencies not found at all, or not existing to the same degree, in the healthy. The sum of these changes constitutes a class of facts the most important in our investigation, and also to a proper understanding of the condition of the sick. For our present purpose it will only be necessary to consider such of these changes as have reference to impressions from drug agents. In a given case of disease the patient is often found to be preternaturally sensitive to the smallest quantities of some drugs, while there is an equal insensibility even to large quantities of others. This is a common experience. Why is it so? If we are not mistaken, a satisfactory answer to this question will be little less than an exposition of the law of the dose. These changes of susceptibility, then, constitute the first class of the general elements of our problem. Those of the second belong to the drug.

These consist, in general, of that power which belongs to drugs to produce disturbances in the actions of the living forces so that these no longer move in that harmony which preserves the comfort and safety of the individual. It is this power so to act that constitutes any substance a drug. And it is with this power so to affect living organs, in special conditions of susceptibility, that we have to do in determining the dose in a given case of disease, and also the law which dominates the dose in all cases. That is to say, after having settled the first question, in all cases of prescribing—what is the remedy? this degree of special susceptibility in the organs, in the given case, is just that which decides the next question—how much of this remedy is required to restore the lost balance of the vital forces in that case—which constitutes the whole problem of cure. How can the degree of this special susceptibility to the action of the selected drug be ascertained before its administration? Simply by an extension of the same process of inquiry that resulted in the discovery of the true remedy. The result of that inquiry answered the question, *what is like?* That is, what is the drug, the action of which on the healthy living organism is most like the phenomena of this lost balance, the disease. An extension of the inquiry, *how much is it like?* when answered, determines the quantity of the drug required, this being in the inverse ratio of the similarity. And this we unhesitatingly declare to be the law of the dose as to *quantity* or *potence.*

If this be admitted, as it may be for the sake of examination, the questions which naturally follow are, what is the definite meaning of degree of similarity in this connection, or of the question, *how much is it like?* and how can this degree of similarity be determined? and these questions ought to be answered. In order to a clear understanding of the proposition, we must go back of its announcement, and examine the meaning of its terms. *Like—Similarity.* What do we mean by these terms, when we refer to the remedy and the dose? Evidently that similarity which is the essence of the law of cure which we all recognize. And what is this? We have endeavored to point this out in a previous paper, very briefly. But, in order to a clear statement of the view we wish to present of the law of the dose, we shall be compelled to repeat a part of what was there stated, that we may show its connection with our present subject. In brief terms, then, the like which cures is the resemblance of the characteristic symptoms of the drug to those of the disease. By characteristic symptoms of the drug and the disease is meant those symptoms which belong to each as individuals, and impart to them their individual character, not at all those which belong to these in common with the other members of their class. In the examination of a case of disease with the object of discovering its curative, we shall find presented a class of symptoms which we have met often before, and to the group they compose we have, for convenience, given a name, and this name we use whenever we meet the group, and by this the group is understood to be represented. These are the generic symptoms. A careful examination will discover other symptoms which are not met in all the members of the class; they make no part of the defining group, and perhaps, indeed very likely, some of them have been found only in the case under examination. These are the specific, or characteristic symptoms. In a case of dysentery, for example, the frequent, painful discharges of blood or of bloody slime, with tenesmus and fever, are symptoms common to the members of the class we call by that name, while pains extending to the back, pain and tenesmus ceasing, for the time, with the discharge, the pain in the back, more particularly in the lower lumbar vertebræ, are symptoms which do not belong to the class but to individuals, and when met are characteristic of those individuals. So, in examining the pathogenesis of drugs, there are found symptoms which are common to many, and some to most drugs. These, of course, cannot be characteristic of any individual. How many drugs will cause pain in the head, loss of appetite, thirst, diarrhœa, vomiting, etc. These, with drugs, are the equivalents of the generic symptoms of the disease. Of the many drugs which cause pain in the head, but one, so far as I know, causes a violent, throbbing pain, with sense of fullness and distention as if the head would burst, turgid redness of the face, and all aggravated intensely by the slightest motion. The loss of appetite is only in some cases accompanied by loathing, or by nausea, or it may disappear on tasting food, or it may be only in relation to particular kinds of food. The thirst may be for cold drink or warm; for large or small quantities at a time; for drink at long or short intervals; or it may be for only particular kinds of drink; or be limited to some particular hours of the day or night. The diarrhœa may be watery, slimy, feculent, or mixed; the discharges excited by various causes, and accompanied by very different phenomena, and occur most frequently at or be limited to, certain hours in the twenty-four. The vomitings may be of substances of very different character, accompanied by different phenomena; aggravated or relieved by different circumstances. These are the equivalents of the specific or characteristic symptoms of disease. These are the elements which characterize the action of different drugs, and so enable us to select that which is the most certain cure in a given case. It is the likeness of these specific symptoms of the drug to the specific symptoms of the disease which the law of cure demands, while resemblance in those symptoms which are common to the class is of very little worth as indicative of the specific remedy.

With this view of the law of cure, and this explaining of the term characteristic or specific (we use the terms interchangeably), we are prepared to answer, first, the question, *What is the like which cures?* The similarity of the characteristic symptoms of the drug to those of the disease, and not at all of those which are generic. And, second, what we mean by the question, *How much is it like?* How great is the *number* of the characteristic symptoms of the disease which find their counterpart in those of the drug selected as the curative, and how exact is the resemblance of those of the one to those of the other. By a comparison of the two classes, in these two particulars, we learn the degree of resemblance which is undoubtedly the exponent of the law of the dose, which we have declared to be, as to the quantity of the drug, or potence, inversely as the similarity of these two classes of characteristics. That is, the greater the number of the characteristic symptoms of the disease found represented by similars in those of the drug, the less quantity (higher potence) of that drug is required for the cure. The degree of the exactness of similarity of these symptoms, of course, enters into the account in determining the question of compliance with the law of the dose as here declared.

The whole relationship of drugs, as curatives, to the diseases which afflict our race, exists in the one fact of susceptibility. If the drug be without power to affect the disturbed actions of the vital forces of the patient, it can have no power to cure. If in the patient there be wanting a susceptibility to its impressions, this relationship, as to this drug, does not exist. On what, then, does this susceptibility depend? On this very similarity of those elements of the disease, which declare its specific nature, to those which are distinctive of the drug. And the degree of susceptibility must, it seems self-evident, be in the direct ratio of the degree of this similarity. In perfect health a man may swallow one or more grains of Ipecac. without danger, or discomfort, possibly. But if the same man be suffering from violent dyspnœa with sense of constriction of the throat; tickling which extends from the bifurcation of the bronchi to their extremities, exciting violent spasmodic cough, cold dampness of the skin, cold sweat on the forehead, and restlessness which compels to violent tossing from place to place, and finds rest or relief in none; he will realize such effects from a much smaller dose as will be quite conclusive of a difference of susceptibility to the action of this drug, when he is, from other causes, experiencing sufferings so characteristic of its action. A quantity much smaller than might be swallowed with impunity in health, in these circumstances might be speedily fatal, certainly would leave in the patient slight disposition to repeat the experiment. The difference is merely one of susceptibility, and this is but the necessary result of the similarity we are discussing. In case of a patient presenting this group of symptoms, the merest tyro in prescribing could not hesitate as to the remedy. There is but one. And now, what shall be the dose? Let him apply the law we have declared, and he will have no reason for dissatisfaction. The resemblance of the group to the characteristics of the drug is great, and therefore by the law, if he prescribes best, he will give a small quantity, *i. e.*, a high potence rather than a low, and the result will justify the practice. Indeed, in such a case we have no doubt the cure would be prompt, even from the highest of those which have been employed, nor that the cure would be more speedy and complete from this than from a lower number.

But, instead of this group, suppose we find great dyspnœa with hot, dry, turgid skin; restless agitation and loud complaining; a sense of fullness and pressure in the chest, which seems to prevent the air from entering the lungs; a tensive pain across the forehead just above the eyebrows; we have a group differing in its characteristics from the first supposed, though it would be called by the same name. In this Ipecac. would not be so dangerous in moderate quantities nor curative in any quantity. The whole group would be called asthma in both cases, and yet in their curative relationship they have nothing in common. This difference it is which constitutes our guide in the selection of our curatives; while the degree of resemblance of the characteristics as explained above decides as to the dose. But why would not Ipecac. be hurtful or curative in this group? Because of the absence of that similarity which is the essence of curative susceptibility to the action of drug agents upon the sick.

If it be objected to this exposition of the law of the dose, that the difficulty of its practical application is great, because of the required intimate knowledge of the materia medica, and therefore its truth is improbable, or its practical value of little worth, the difficulty is admitted, while the conclusion drawn from it is denied. We can see no good reason why this should not be met like other difficulties, to be overcome. The difficult application of a law can neither disprove its existence nor effect its repeal.

If we have been successful in so stating the principles of the law of the dose as to make them and their application understood, we believe it will be plain at once why it is that cases are occasionally met in which high potencies cure where low have failed, and the reverse. The explanation is found in the degree of susceptibility of the patient to the action of the drug, and this is in the direct ratio of the similarity of the characteristics of the drug and the disease. Where the susceptibility is great and the quantity of the drug relatively great, it is not difficult to see how its direct action, being so like to the action of the disease it is intended to relieve, may so, by this action, oppress the vital forces as to render them incapable of a curative response; or so intensify the diseased action, as to increase pain and danger, without any corresponding curative effort on the part of these oppressed forces; while, on the other hand, where the quantity (we use the word here as equivalent to potence) is adapted to the susceptibility of the patient, this evil and disappointment are avoided, and the best result secured.

In conclusion, we earnestly urge the attention of practitioners to that study of the materia medica which will make them familiar with the science in its characteristics. If this be difficult, this is no reason, it is submitted, why the difficulty should not be manfully met and overcome. We also urge the importance of the law which we have attempted to discuss as a means of extinguishing the party strifes which prevail too much on this subject of the dose, and which now are only a source of evil to our school of medicine. If the dose be a matter of law, let this be known, and strife on its account must cease. Put the matter to practical test and see if great similarity of characteristics does not prove great susceptibility to drug action, and great susceptibility is not best met and cured by high numbers or small quantities, as it should be by this law.

